# DNA Vaccination in Farmed Fish with a Focus on Salmonid Alphavirus Infection in Atlantic Salmon (*Salmo salar* L.) in Norway

**DOI:** 10.3390/v18060639

**Published:** 2026-06-03

**Authors:** Espen Rimstad, Øystein Evensen

**Affiliations:** Faculty of Veterinary Medicine, Norwegian University of Life Sciences, P.O. Box 5003, N-1432 Ås, Norway

**Keywords:** fish viruses, DNA vaccines, Salmonid alphavirus

## Abstract

Pancreas disease (PD), caused by Salmonid alphavirus (SAV), is a notifiable disease in Atlantic salmon (*Salmo salar* L.) in Norway. Conventional inactivated virus vaccines have shown variable effects in mitigating the disease, and a DNA vaccine has been used over the last 7–8 years, which may have resulted in the reduction in the number of reported PD cases. This manuscript provides a comprehensive overview of DNA vaccination in farmed fish, with a focus on the licensed DNA vaccine, Clynav^®^, against SAV3 infection. It explores the biological underpinnings of SAV infection, immune mechanisms activated by DNA vaccines, and the benefits and limitations of this approach. Although antigen processing and presentation mechanisms following DNA vaccination in fish remain incomplete, studies document robust innate responses and measurable adaptive immunity, including neutralizing antibodies, as seen in Clynav, and transcriptomic studies indicate that cell-mediated immunity is evoked under experimental conditions. Comparative trials demonstrate that DNA vaccination reduces viral load, tissue pathology, and, potentially, viral transmission, outperforming traditional oil-adjuvanted vaccines. Additionally, DNA-vaccinated fish show improved growth performance under field conditions. These findings support DNA vaccination as a promising strategy for controlling PD in salmon aquaculture, with implications for fish health, welfare, and sustainable production.

## 1. Introduction

Pancreas Disease (PD) of farmed Atlantic salmon (*Salmo salar* L.) and rainbow trout (*Oncorhynchus mykiss*) is caused by Salmonid alphavirus (SAV), now renamed Alphavirus salmon. Fish with acute PD are typically lethargic and initially exhibit pancreatic tissue inflammation, followed by inflammation of the heart and skeletal muscles [1]. In Norway, systematic and comprehensive testing, rigorous management, and vaccination with inactivated virus vaccine in PD enzootic areas have not efficiently reduced the number of outbreaks [2,3].

**Figure 1:**
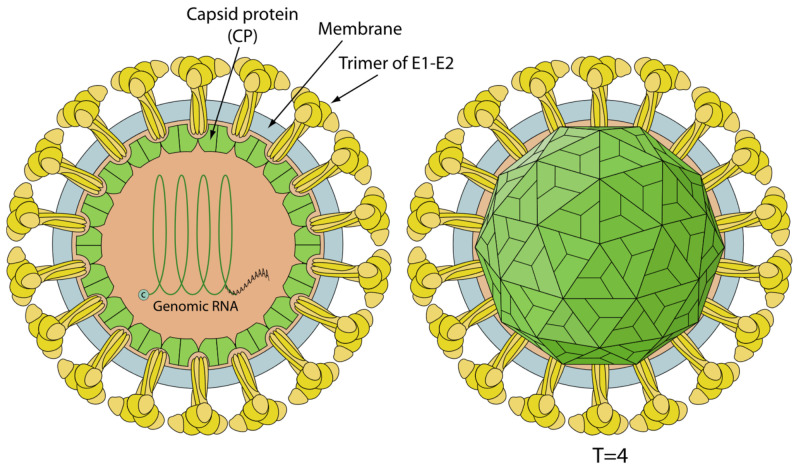
Alphavirus particle, showing the trimeric E1-E2 spikes, icosahedral capsid, and genomic RNA (Source ViralZone, SIB Swiss Institute of Bioinformatics).

SAV is an enveloped, spherical particle (Figure 1) with a single-stranded positive-sense RNA genome. The genome encodes five structural proteins (capsid, E3, E2, 6K, and E1), the building blocks of the virus particle, and four nonstructural proteins (nsP1-4) mediating the viral RNA transcription and replication [4,5]. The surface of the virus particle is composed of spikes formed by the glycoproteins E1 and E2 [6]. SAV has been categorized into subtypes (SAV 1–SAV 7) based on nucleic acid sequences of E2 and nsP3 [7]. Antigenic variation among genotypes is considered low, as monoclonal antibodies raised against a particular SAV genotype are likely to cross-react with other SAV isolates [8,9]. SAV is known to cause diseases in salmonid fish but has also been isolated from non-salmonid marine fish species [7].

The glycoproteins E1 and E2 (“E” stands for envelope) for alphaviruses are embedded in the viral envelope and make most of the antigens on the outer part of the virion. Each spike consists of a trimer of an E1/E2 dimer, and the main functions are binding to cellular receptors and penetration into the cytoplasm. E2 binds to cellular receptors, allowing the virus to enter endosomes. In the endosome, the virus is exposed to a low pH, which changes the conformation of the E1 protein, exposing a hydrophobic peptide that inserts into the endosomal membrane and mediates fusion of the endosomal membrane with the viral envelope. This fusion releases the viral nucleocapsid into the cytoplasm, where it disassembles, and the genomic viral RNA is released and translated, thereby initiating infection [10].

The E2 protein is a primary target of antibodies that prevent virus particles from attaching to cells (i.e., neutralizing antibodies), and in a vaccine, this protein will be an important component [8,10].

The environmental temperature is crucial to the duration of SAV’s infectivity outside the host. Laboratory tests indicate that SAV can remain infectious in aquatic environments for up to 65 days, but this period is inversely related to temperature [9]. SAV is rapidly inactivated at 60 °C, high UV radiation, or extreme pH levels. The presence of organic matter reduces the effectiveness of disinfectants [9].

### 1.1. PD Disease Signs and Pathological Changes

The clinical signs of acute PD can be briefly described as sudden inappetence, lethargy, and inability to maintain position in the water column [11]. The histopathological changes primarily consist of pancreatic necrosis and myopathy in the heart and skeletal muscles [8]. There is a 1-week lag between the onset of acute pancreatic necrosis and the onset of clinical signs [12].

### 1.2. Transmission

Atlantic salmon and rainbow trout are both highly susceptible species to SAV infection. Horizontal transmission through water and direct or indirect contact between fish have been observed in studies showing transmission from shedder fish to naïve cohabiting fish, including proven transmission between farming sites and the virus being spread via water currents; transmission has also been found indirectly through phylogenetic analyses [13,14,15,16,17,18]. Long-distance transmission into free areas is often due to the movement of infected live fish [15,19]. The presence of SAV in fat leaking from dead fish may also contribute to waterborne spread [17]. SAV has been detected in salmon lice during outbreaks [20], but transfer of SAV from lice to susceptible fish has not been reported. However, it is worth noting that in mammals, alphavirus transmission occurs through blood-feeding arthropods, primarily mosquitoes.

While vertical transmission of SAV, meaning transmission from one generation to the next, has been suggested for Atlantic salmon [21], other studies show no evidence to support this [19,22]. A risk assessment conducted by the Norwegian Scientific Committee for Food Safety concluded that under farming conditions that comply with the Food Safety Authority’s disinfection regulations, the risk of vertical transmission of SAV is negligible [23].

Good husbandry practices, such as segregation of generations, fallowing between insets, stocking with high-quality smolt, removing dead fish, and reducing stress during handling, help minimize the risk of SAV infection and clinical outbreaks. The prevalence of SAV infection in a farmed Atlantic salmon population varies with the course of infection, with high prevalences (70–100%) observed during clinical disease outbreaks [24].

### 1.3. Detection of SAV- WOAH Criteria

SAV infection is a notifiable to the World Organization of Animal Health (WOAH), i.e., not only clinical outbreaks of PD but also the SAV infection itself. WOAH has criteria for listing diseases, among which are: (1) the international spread of the pathogenic agent (via aquatic animals, aquatic animal products, vectors, or fomites) is likely; (2) a precise case definition is available; and (3) a reliable means of detection and diagnosis exists.

The WOAH recommends collecting heart and mid-kidney samples to detect SAV. During an outbreak, the heart usually contains more SAV than other tissues and is therefore the preferred organ for sampling. Approximately three weeks after SAV infection, blood serum or plasma is suitable for detecting specific SAV antibodies in a virus neutralization test [25].

The presence of infection with SAV is considered to be confirmed if one or more of the following criteria is met: (i) A positive result on tissue preparations by conventional RT-PCR and sequencing of the amplicon; (ii) SAV-typical CPE in cell culture followed by virus identification by conventional RT-PCR and sequencing of the amplicon; and (iii) a positive result on tissue preparations by immunohistochemistry and by conventional RT-PCR and sequencing of the amplicon. These requirements are identical for both apparently healthy and clinically affected fish. The WOAH requirements for confirming an SAV infection do not focus on the presence of typical histopathological lesions.

There is a difference between the national criteria for PD diagnosis, in which confirmation of a disease outbreak is sought based on the detection of typical histopathological findings and virus detection, typically by RT-PCR, and the WOAH criteria. In 2024, 43 of the 48 PD cases in Norway were based on histopathological changes compatible with PD and the detection of SAV in the same individual [26].

### 1.4. The Trend for PD in Norway

There are two separate PD epidemics in Norway: one caused by SAV3, a SAV subtype detected only in Norway, and the other by SAV2. There is little overlap in the geographic areas where these two epidemics occur.

**Figure 2:**
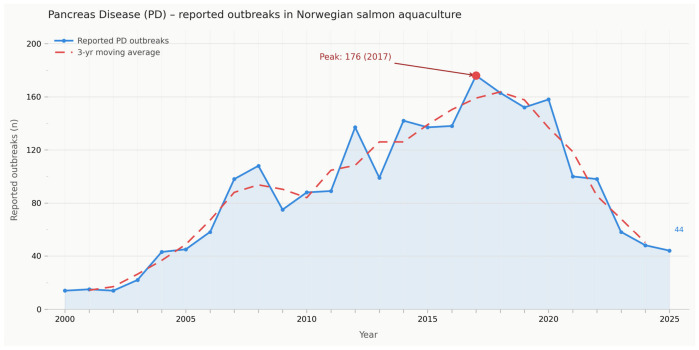
Number of reported PD cases in Norway (*y*-axis) over the period 2000–2025. Numbers are given as the combined SAV2 and SAV3 cases (prepared by the authors).

The number of PD cases listed in the annual Fish Health Report of the Norwegian Veterinary Institute includes both suspected and confirmed PD cases. Cases of suspected or confirmed PD from the preceding year that remain in the sea are not included in the dataset used to calculate the number of PD cases for the coming year. This implies that the number of infected populations in a PD zone at a particular point could be affected by the number of infected fish populations from the previous year that may still be present. There has been a general trend since 2020 toward fewer PD outbreaks. In 2024, 48 new PD cases were recorded (15 SAV2 and 33 SAV3) [26], representing a significant reduction from 2020, when 158 cases were reported (Figure 2).

## 2. DNA Vaccination of Fish

DNA vaccination technology is rooted in gene therapy. More than 30 years ago, it was shown that injecting naked plasmids into the muscles of mice could elicit an immune response [27]. Intramuscular injection of a plasmid encoding a gene of interest will result in a transient expression sufficient to evoke an immune response. The efficacy of DNA vaccines has been documented for several fish-pathogenic viruses [28,29,30] and bacteria [31], but studies on bacteria remain mostly experimental. More specifically, it has been demonstrated that DNA vaccines induce protective immunity to several viral infections in fish, rhabdoviruses of rainbow trout and Atlantic salmon [32,33], channel catfish herpesvirus infection [34], red sea bream iridovirus [35], and pancreas disease, salmonid alphavirus infection, in salmonids [30]. DNA vaccines, with a few exceptions [36,37], rely on intramuscular injection to induce protective immunity. Oral DNA vaccines induce protective immunity against infectious pancreatic necrosis (IPN) in trout [36,38]. In contrast, a low level of protection has been documented at early post-oral vaccination time points against IHN [39]. The distribution to internal organs following intramuscular (i.m.) injection has not yet been studied in detail. However, it has been shown that a luciferase-encoding plasmid can be distributed to internal organs, and gene expression can be detected in organs shortly after administration [40]. Furthermore, luciferase expression in internal organs has been observed over an extended period (up to 24 months) [41], and protection has been documented up to 78 weeks post-vaccination [42].

## 3. Mechanisms of Immune Induction by DNA Vaccines and How to Assess Them

DNA vaccines are generally composed of plasmids engineered with bacterial and eukaryotic elements that enable transgene expression in vertebrate cells [43]. They are inherently safe, non-replicating, and generally well-tolerated in vaccinated animals [44]. Regulatory considerations and safety aspects of DNA vaccines have been reviewed earlier [45,46]. DNA vaccines encode immune-protective antigens derived from pathogens such as viruses or bacteria, aiming to trigger a targeted immune response. This response can protect the vaccinated animal from disease and reduce mortality. A key challenge in DNA vaccine design is selecting antigens that induce protective immunity, particularly those that elicit neutralizing antibodies and activate effector T cells. In fish, commercially available DNA vaccines are delivered by intramuscular injection.

Several strategies have been attempted to improve the potency of DNA vaccines. This includes the use of efficient promoters, codon optimization, addition of adjuvants, electroporation, intradermal delivery, reduction of extracellular DNA degradation, and prime–boost strategies [47].

### 3.1. In Vitro Expression of the Transgene

**Figure 3:**
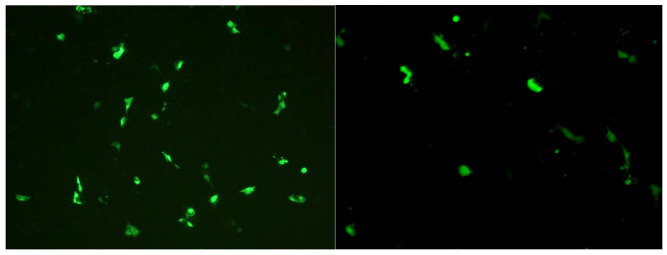
Expression of IPN virus VP2 protein, stained with rabbit antiserum to IPNV (**left**). Pmax-Green Lantern expression (model antigen, (**right**)). Photo: Ø. Evensen.

In DNA vaccine construction, strong eukaryotic promoters are typically chosen to ensure efficient expression of the encoded antigens. Plasmids contain essential elements besides the promoter region, an antigen-coding sequence, and regulatory elements necessary for proper antigen expression [48]. The expression of the gene from the prepared plasmid can be tested by in vitro transfection in permissive cell lines (Figure 3), and in vitro protein expression is a good starting point for further in vivo testing [49].

In vitro expression provides valuable information about the functionality of the prepared construct and can be used to document cellular responses to the antigen in question, including any cell toxicity. Such studies can document cytoplasmic or membrane-associated expression of encoded antigens.

### 3.2. Mechanisms of Immune Induction

**Figure 4:**
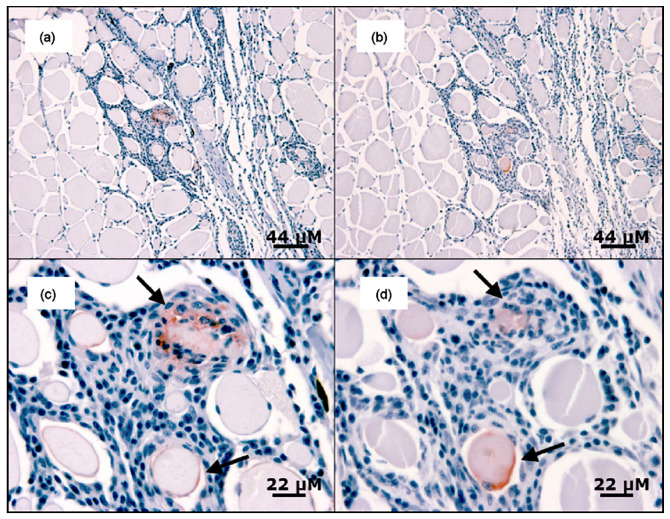
Expressed antigens following immunization with a plasmid encoding (**a**,**c**) VHSV G protein in muscle cells (arrows) or (**b**,**d**) IHNV G protein in muscle cells (arrows) of rainbow trout in the same cells [52].

Fish vaccinated by the intramuscular (i.m.) route will take up the plasmid in skeletal muscle cells (Figure 4), and the expressed antigen will be found in the sarcoplasm or presented on the cell surface. This is well documented for DNA vaccines encoding the G protein of viral hemorrhagic septicemia virus (VHSV) and infectious hematopoietic virus (IHNV, Figure 4). It is believed that the high efficacy of vaccines used to protect against infection with the two Novirhabdoviruses is attributable to the surface localization of the G protein in transfected muscle cells. DNA vaccines against IHN or VHS provide a relative percent protection (RPS) of >80, with mortality approaching 100% in non-vaccinated control fish under experimental conditions [50,51].

Studies of DNA vaccines that encode surface proteins (capsid proteins) of naked viruses in fish have yielded lower levels of protection, as seen with DNA vaccines tested against infectious pancreatic necrosis virus (IPNV) [53], or with no protection at all against nodavirus infection in turbot (*Scophthalmus maximus*) [54]. There is some variation in this concept, providing the polyprotein gene of IPNV and thus ensuring proper cleavage of the protein, decreased the viral load after IPNV infection compared to controls [55]. It has been shown that an orally delivered DNA VP2-encoding vaccine elicited strong protection against a lethal challenge in rainbow trout, in which mucosal responses may play a role [38,56]. Furthermore, a heterologous vaccine encoding the G protein of IHNV and the VP2 protein of IPNV conferred high levels of immunity in rainbow trout against both viruses, as evidenced by protection against IHNV mortality and a reduction in IPNV viral load [57]. The mechanisms underlying an improved efficacy for a combined IHNV-IPNV vaccine are not understood. In the study referenced, the VP2–VP3 genes were cloned downstream of the G gene in the pcDNA-IHN vector to produce a bivalent pCh-IHN/IPN DNA vaccine [57]. The authors did not provide documentation of joint protein expression of G and VP2-VP3 proteins in transfected cells, and a better understanding of whether VP2-VP3 was translocated to the muscle membrane would have been of interest. Double expression of VHSV and IHNV G proteins in the same muscle cells was observed after simultaneous i.m. injection when the two G proteins were encoded by different plasmids (Figure 4); protection against both infections was also documented by others [28].

**Figure 5:**
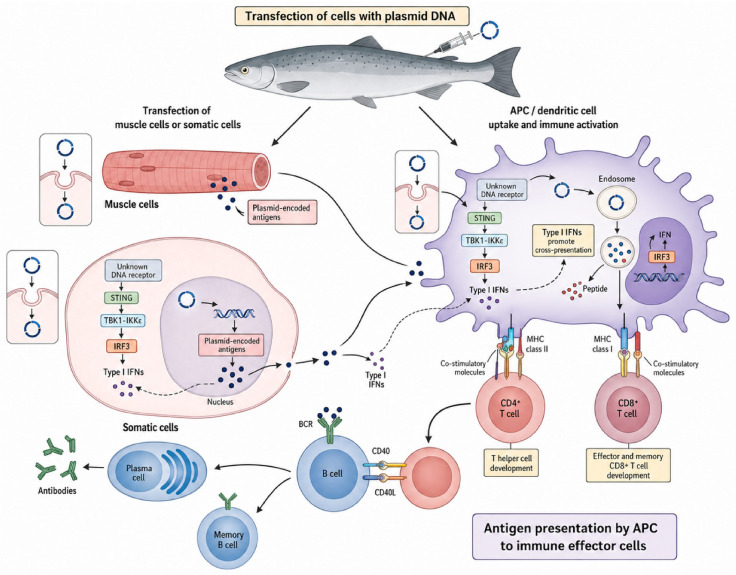
Schematic overview of steps in the induction of immunity following DNA vaccination. Plasmids are taken up in muscle cells, somatic cells, or directly in APCs. Antigen is expressed in muscle and somatic cells and can be secreted to neighboring APCs for subsequent presentation via MHC-I or MHC-II. APCs that take up plasmids directly present antigen via MHC-I to CD8^+^ T cells or via MHC-II to CD4-positive cells, with subsequent CD4 help provided to B cells (adapted from [58] and prepared by Ø. Evensen).

The current concept of immune induction by DNA vaccines is summarized in Figure 5. It includes either plasmid uptake by professional antigen-presenting cells (APCs) or plasmid uptake, antigen expression, and secretion from somatic cells, followed by subsequent uptake by professional APCs (Figure 5).

The process outlined in Figure 5 has not been proven following DNA vaccination in fish, and the exact mechanism by which immunity is induced (in fish or higher vertebrates) remains unknown. APCs have been described for rainbow trout in the skin [59], but any presence in intermuscular tissue has not been shown. Descriptive studies have profiled the transcriptome following DNA vaccination in Atlantic salmon [60], indicating enrichment of antigen presentation pathways. Still, no documentation of any functional responses was presented. The understanding is that expression of the transgene, with subsequent protein production by the somatic muscle cells, will elicit an inflammatory response in the intermuscular interstitium surrounding the cells, near the site of foreign antigen expression. The cells infiltrating the area are lymphocytic, assessed by light microscopy.

The diverse and complex cellular immune mechanisms are not fully understood (Figure 5). Plasmids are likely taken up (directly) by antigen-presenting cells, or, when antigens are secreted/released from somatic cells, by APCs that then take them up. Subsequently, APCs interact with the humoral and cellular arms of the immune system. The extent to which somatic cells secrete the same antigens for subsequent uptake by antigen-presenting cells for expression of foreign antigens is not well understood in fish or other vertebrates. It is important to note that antigens expressed on somatic cells do not activate cytotoxic T cells via MHC-I peptide presentation. This can only be achieved by presenting T-cell antigens from professional APCs in conjunction with several APC-specific co-stimulatory molecules, as observed in mice [61,62]. Further, muscle cells in fish do not normally express MHC-II [63], so presentation to CD4 or B cells is not expected. The potential involvement of cross-dressing has not been studied following DNA vaccination, but has been documented under various infections [64,65].

**Figure 6:**
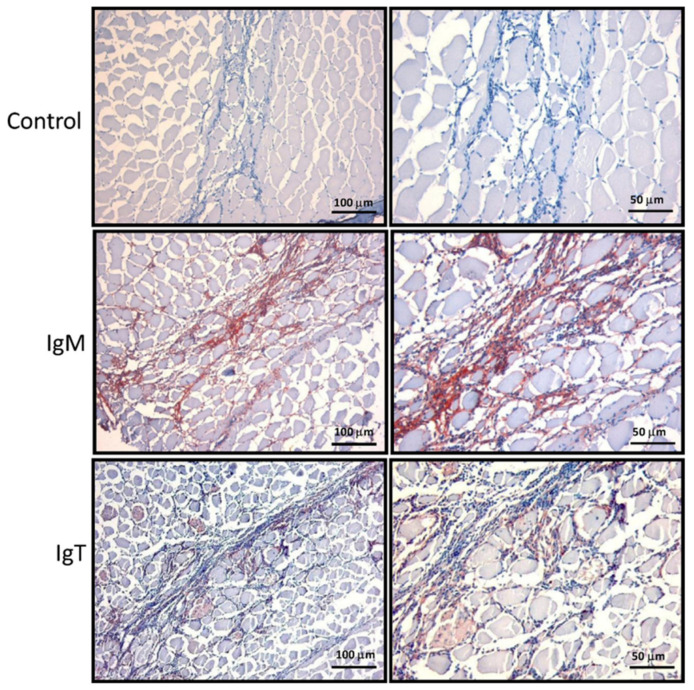
Muscle from DNA-vaccinated trout using immunohistochemistry for the detection of IgM^+^ or IgT^+^ cells in areas where the plasmid has been injected. The reddish color indicates a positive reaction [66].

Studies of local muscle responses to DNA vaccination against VHSV infection have shown a high abundance of Ig-positive cells in inflamed areas, including both IgM- and IgT-positive cells [66]. The authors link the presence of B cells to increased chemokine expression in areas where the DNA vaccine was injected. It is, however, not known whether the B cells are activated to differentiate into plasma cells and produce subsequent Ig, although the diffuse response in the intermuscular tissue would indicate local release of immunoglobulins into the surrounding tissue (Figure 6).

While the exact mechanisms of antigen presentation are not known, there is substantial evidence that antigens on muscle surfaces that elicit a strong local inflammatory response [52,66,67] (Figure 4) correlate with the induction of an immune response. This has been studied for VHSV, and it is well documented that localization of the G protein to the cell membrane of muscle cells is associated with measurable (protective) immune responses.

**Figure 7:**
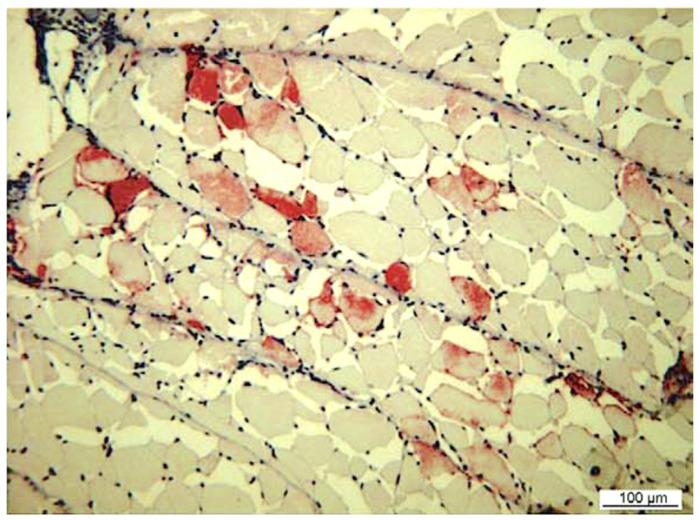
Immunohistochemical staining for N-protein (from the VHS virus) showed no or minimal inflammation in areas with high protein expression [67].

Moreover, at early post-vaccination stages, protective responses are linked to strong innate responses, IFN-I, and downstream Mx, amongst others [68,69]. Later, vaccinated fish raise adaptive immune responses with long-term efficacy and protection [69]. In contrast, plasmid constructs expressing the nucleoprotein (N-protein) of VHSV in transfected muscle cells elicit mild inflammatory responses (Figure 7), and antibodies are not formed or are present at very low levels. 

Boudinot and coworkers were the first to study host gene expression at the injection site following DNA vaccination against VHSV and IHNV infection [28]. They observed an increased expression of Mx mRNA and MHC class II genes by (RT)-PCR at the injection site. In a similar study, Takano and coworkers [70] showed up-regulation of MHC and T-cell receptor mRNA in muscle tissue of Japanese flounder 1 day after DNA vaccination against hirame rhabdovirus infection. Boudinot and co-workers [28] also showed expression of viral G protein at the injection site, and Lorenzen and co-workers [67] found Ig^+^, complement factor 3 (C3), and MHC II-positive cells by immunofluorescence close to VHSV G protein-positive muscle cells. In line with what is mentioned above, MHC-II^+^ cells are infiltrating cells, not myocytes per se [67], likely macrophages or B cells that infiltrate the area where muscle cells express the foreign antigen.

In summary, following DNA vaccination in fish, the immune response is initiated primarily at the intramuscular injection site, where the plasmid is taken up by skeletal muscle cells, which express the encoded antigen either within the sarcoplasm or on the muscle cell surface. This is best demonstrated by vaccines targeting viruses such as VHSV and IHNV, where surface expression of the G protein is associated with strong protective immunity. In contrast, DNA vaccines encoding internal or capsid proteins, such as those from IPNV or nodavirus, tend to elicit weaker or no protection. Interestingly, bivalent vaccine constructs combining protective and non-protective antigens—for example, a vaccine encoding the IHNV G protein and IPNV VP2—can still induce immunity against both pathogens. The exact process of immune induction is not fully understood and may involve either direct uptake of the plasmid by professional antigen-presenting cells (APCs) or indirect uptake of antigens from expressing muscle cells, possibly secreted or released by muscle cells and then processed by APCs [71]. Muscle cells do not express MHC-II and cannot directly activate helper T cells or B cells, but surrounding infiltrating cells, such as macrophages and B cells [67], may act as APCs. At early post-vaccination stages, protection is linked to innate immune responses, including the activation of interferon pathways and related genes, such as Mx [68]. Over time, adaptive immunity develops, resulting in long-term protection. These findings indicate robust immune activation following DNA vaccination; further research is needed to elucidate the specific cellular interactions and functional outcomes underlying DNA vaccine-induced immunity in fish.

### 3.3. Documentation of Elicited Adaptive Immune Responses Post-Vaccination

The assessment of protective immunity for a fish DNA vaccine is best performed using vaccination and in vivo challenge experiments. For viruses that cause mortality following experimental challenge, the direct assessment of vaccination effects is straightforward. For non-lethal virus species, such as infectious pancreatic necrosis virus, the impact of vaccination is assessed by measuring virus load [57] and/or protection against pathology in target organs [72].

The specific adaptive responses following DNA vaccination include antibody responses to rhabdoviruses [28,73] and SAV [30,74]. Circulating neutralizing antibodies have been detected as early as 23 days post-vaccination against IHN and VHS [28], and the onset of immunity with Clynav is at 399 degree-days [75]. In general, neutralizing antibodies, while measurable, can vary between vaccinated fish [76]. Antibody levels will increase with the number of post-vaccination degree days [69,77]. Still, protection against mortality has been observed in the absence of detectable circulating neutralizing antibodies [50,51,77,78] or with low post-vaccination levels of neutralizing antibodies [30]. Studies indicate that the challenge virus will boost humoral responses, as evidenced by high post-challenge levels of neutralizing antibodies [79], but how the DNA vaccination elicits the boost response remains unclear, since high levels of neutralizing antibodies are also observed in naïve (non-vaccinated)/challenged fish [79].

### 3.4. Antibody Responses After DNA or Inactivated Whole-Virus SAV Vaccination

**Figure 8:**
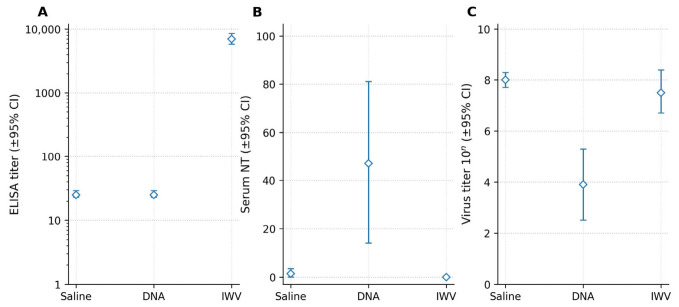
DNA- and IWV (inactivated whole-virus)-vaccinated fish plus saline controls. IWV-vaccinated fish have high levels of circulating (**A**) antibodies by ELISA, but (**B**) no neutralization is observed, while DNA-vaccinated fish have low and variable levels. Serum neutralizing titer (NT) correlates with the ability to control viral replication (**C**), with virus titer shown for each group [76].

Figure 8 summarizes a study in which a multivalent, inactivated whole-virus (SAV) vaccine, no longer commercially available, was compared with the DNA vaccine against SAV3 (Clynav^®^), and the material originates from both field and laboratory studies [76]. Plasma samples were collected at >750 degree-days post-vaccination and screened for antibody titers by ELISA (using SAV3 as the coating antigen) and for neutralizing antibody titers in cell culture. The laboratory study also included a challenge study (>750 degree-days post-vaccination), and viral levels were profiled in Atlantic salmon heart tissue 19 days post-challenge. Saline (control) was included in all experiments. Virus titers and neutralizing antibody titers were determined as described [74]. The profiles of ELISA antibody levels, neutralizing antibody levels, and viral titer in the heart are shown in Figure 8.

The IWV (inactivated whole-virus) vaccine induces high levels of circulating antibodies, as measured by ELISA, whereas the DNA vaccine induces low levels. In contrast, VNT was not detected in IWV-vaccinated fish, while low levels were found in the DNA-vaccinated group (Figure 8). Interestingly, post-challenge viral levels in the IWV group did not differ from those in the saline group but were reduced by 4 log_10_ in the DNA-vaccinated fish. While the setup is not directly comparable, it suggests a difference in the neutralizing antibody response between DNA and IWV vaccines.

### 3.5. Cell-Mediated Responses Post-DNA Vaccination

Specifically, cell-mediated immune responses are challenging to study in fish due to a lack of appropriate models (e.g., isogenic or MHC-matched fish). Cell-mediated immune responses following DNA vaccination have been studied in rainbow trout vaccinated with a VHSV-G DNA vaccine [80] and in isogenic rainbow trout vaccinated with an inactivated whole-virus vaccine against salmonid alphavirus infection [81]. In the former study, peripheral blood leukocytes killed MHC-I-matched RTG-2 cells and VHSV-infected xenogeneic cells. The specificity of the response was confirmed using VHSV-infected target cells that were not killed [80]. The killing of MHC-I-matched and xenogeneic cells indicates the involvement of cytotoxic cells and natural killer cells. For the latter study, no strong cytotoxic response was found [81]. No studies are available to document the contribution of cell-mediated immunity to the Clynav vaccine.

### 3.6. Modalities of Delivery

There are few studies on the impact of administration modalities for DNA vaccines for fish. One study included intradermal delivery of an IHN-DNA vaccine, as well as alternative routes of administration, such as intramuscular and intraperitoneal [82]. This was carried out in rainbow trout fry, and the findings were that the intradermal route was as efficient as the intramuscular route in inducing immunity. Intraperitoneal injection fell short compared to the other routes [82].

### 3.7. Duration of Immunity

The duration of antigen expression at the injection site depends on various factors, including the stability of the DNA vaccine, the efficiency of cellular uptake and expression, and the turnover rate of host cells. Generally, DNA vaccines can induce antigen expression for a limited period, typically several days to weeks after administration. However, booster doses may be required to sustain long-term antigen expression and immunity. The duration of immunity for the plasmid vaccine against pancreas disease in Atlantic salmon (Clynav^®^) is 9.5 months for the reduction of mortality and 12 months for impaired daily weight gain and cardiac, pancreatic, and skeletal muscle lesions (according to the product leaflet) [75].

The extent to which the duration of immunity depends on the persistence of the plasmid in internal organs, particularly immune organs, is unknown. The ability of vaccine DNA to persist in host cells and tissues depends on factors such as the vector’s nature, the immunogenicity of the encoded antigens, the host’s immune responses, and, likely, the administered dose. In some cases, a DNA vaccine may persist for an extended period within host cells, contributing to prolonged antigen expression and immune stimulation, as found in mice using integrating plasmids, where persistent expression produced sustained antigen production and enhanced CD8+ T-cell response [83].

## 4. DNA Vaccines Against PD Virus Infections

Classically, the defining pathological feature of PD is pancreatic tissue necrosis. Several studies have demonstrated that DNA vaccination can reduce the extent of pancreatic tissue destruction, both under experimental conditions [30,79,84] and in field settings [85]. The reduced but incomplete histopathological changes observed in vaccinated fish suggest that DNA vaccines confer partial protection against SAV infection. Experimental challenge studies also indicate that this protection is non-sterilizing; however, DNA-vaccinated fish consistently exhibit significantly lower viral RNA loads in blood and heart, as measured by RT-qPCR, and reduced histopathological scores in key target organs compared to those given oil-adjuvanted inactivated vaccines [79]. Thus, several independent investigations have reported reduced viremia in DNA-vaccinated groups following SAV challenges, indicating reduced viral burden. It has been speculated that this effect is linked to the enhanced immunogenicity of DNA vaccines, which have induced higher titers of neutralizing antibodies compared to oil-adjuvanted vaccines [30,79].

The impact on mortality using the DNA vaccine versus an oil-adjuvanted inactivated whole-virus (IWV) vaccine has been addressed under field conditions. In one study, the DNA vaccine reduced mortality during PD outbreaks by 1.3% (a statistically significant difference) compared with unvaccinated controls. In contrast, oil-adjuvanted inactivated vaccines did not give statistically significant protection [85]. In another study comparing the effects on growth and mortality in DNA-vaccinated and oil-adjuvanted fish groups, both vaccines improved these outcomes, with the DNA vaccine yielding a more pronounced effect [86]. Two experimental studies comparing DNA and inactivated oil-adjuvanted vaccines reported reduced mortality rates post-challenge (6.4% vs. 10.5% and 9.1% vs. 12.6%, respectively); however, these differences were not statistically significant [74,79].

**Figure 9:**
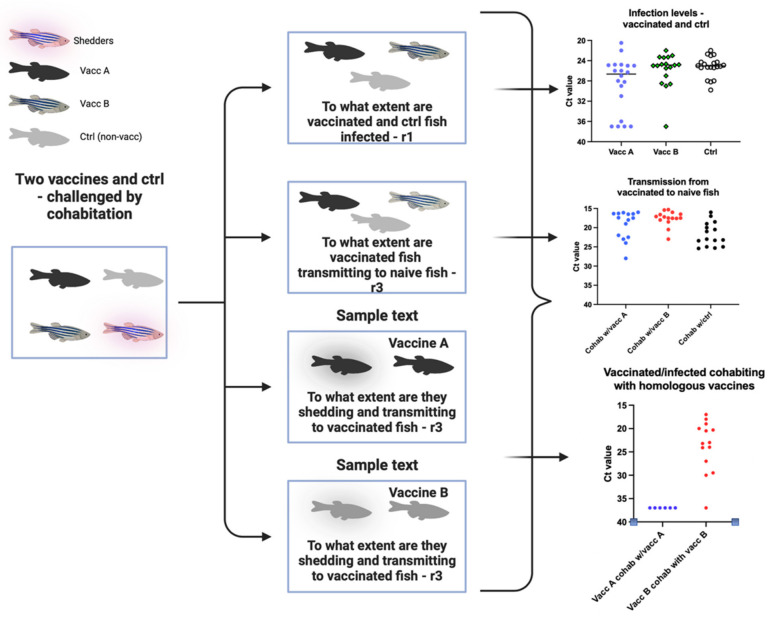
Layout of the experimental setup to study transmission between vaccinated and naïve fish. Vacc A is a DNA vaccine (Clynav), and Vacc B is an IWV vaccine (Alpha Ject micro 1 PD) [79].

Given the lower viral loads observed in vaccinated fish, DNA vaccination may reduce viral transmission. It is well documented that several fish non-DNA vaccines protect against mortality and/or clinical disease, particularly those against bacterial diseases [87]. From an epidemiological viewpoint, it is important to understand the extent to which vaccinated fish become infected and, if so, how much they shed the virus into the environment and into cohabiting vaccinated or non-vaccinated fish. A recent study examined the spread from vaccinated and challenged fish to naïve and vaccinated groups [79]. The experimental setup is shown in Figure 9. While the setup is somewhat complex, the outcome is clear. Two different vaccines, a DNA vaccine and an inactivated whole-virus vaccine (IWV), were used to vaccinate Atlantic salmon. The primary challenge was conducted past the onset of immunity by introducing naïve shedder fish into the challenge tank. The questions raised were: First, will vaccinated fish be infected by the shedder fish, and are there any differences between the vaccines? The upper graph on the right (Figure 9) shows the Ct values for the virus in the heart; in this study, all groups became infected. There is no statistical difference between groups, nor between non-vaccinated controls and vaccinated fish. Then, will vaccinated fish that become infected shed the virus and infect naïve, cohabiting fish? Since SAV infections are non-lethal, the logistics are easier, and, yes, vaccinated and infected fish shed the virus. Naïve cohabiting fish become infected, when DNA-vaccinated fish are shedders and when IWV-vaccinated fish are shedders. The next step was to examine the extent to which vaccinated and infected fish would shed the virus onto cohabiting vaccinated fish. This was done by combining DNA-vaccinated/infected fish with vaccinated fish in the same tank, then sampling at 19 days post-introduction. We did the same for IWV fish. Interestingly: the cohabiting DNA-vaccinated fish transferred to the tank with the DNA-vaccinated/infected fish did not pick up the infection, whereas the IWV groups in the same setup did. It is not known how much virus was shed into the water from the two infected vaccine groups (DNA and IWV), nor whether IWV-vaccinated and infected fish would infect cohabiting DNA-vaccinated fish. These findings show that DNA-vaccinated fish, to a lesser degree, transmitted the virus to naïve cohabitants, and no transmission was observed when DNA-vaccinated fish were added to tanks harboring DNA-vaccinated infected fish [79]. Should this observation also hold under field conditions, it may suggest that population-level vaccination strategies could interrupt the transmission cycle of SAV. Such an effect could partly explain the decline in PD case diagnoses observed after 2020.

Regarding production performance, several studies have documented improved growth in DNA-vaccinated groups. In two field studies, statistically significant increases in harvest weight were found in DNA-vaccinated fish [85,86] and in oil-adjuvanted IWV [86]. Furthermore, in three experimental trials comparing DNA vaccines to oil-adjuvanted inactivated vaccines, significantly higher weight gain was consistently observed in the DNA-vaccinated cohorts [30,79]. In contrast, a recent study evaluating a live attenuated SAV vaccine demonstrated efficacy in reducing viral load and improving survival, but this approach was associated with growth impairment [88].

In summary, current evidence suggests that DNA vaccines against PD may offer several advantages over traditional vaccination strategies, including improved growth performance in vaccinated fish; superior viral clearance, thereby reducing SAV transmission; improved protection against pathological changes; and enhanced induction of neutralizing antibodies.

## Data Availability

No new data were created or analyzed in this study.
